# Assessing the Capabilities of Artificial Intelligence (AI) Tools in Community Medicine: A Comparative Study of ChatGPT, Gemini, and Bing in Community-Based Clinico-Social Case Interpretation

**DOI:** 10.7759/cureus.91917

**Published:** 2025-09-09

**Authors:** Mukesh Shukla, Deepshikha Pandey, Mayank Agarwal, Samarjeet Kaur, Aayushi Goyal

**Affiliations:** 1 Community Medicine, All India Institute of Medical Sciences, Raebareli, IND; 2 Community Medicine, Amar Shaheed Jodha Singh Atayiya Thakur Dariyao Singh Medical College, Fatehpur, IND; 3 Physiology, All India Institute of Medical Sciences, Raebareli, IND; 4 Community Medicine, Ganesh Shankar Vidyarthi Memorial Medical College, Kanpur, IND

**Keywords:** artificial intelligence, bing ai, chatgpt, clinic-social case studies, community medicine, google gemini

## Abstract

Background and objective

Artificial intelligence (AI) is being increasingly integrated into healthcare, offering opportunities to enhance decision-making, education, and patient engagement. Community medicine often involves interpreting clinico-social case studies that combine clinical, social, and environmental dimensions. However, there is limited research evaluating how AI tools perform in analyzing such community-based clinico-social cases. This study aimed to address that gap in the literature.

Methods

A comparative cross-sectional study was conducted using 30 standardized clinico-social case studies covering communicable and non-communicable diseases, maternal and child health, adolescent health, and social pathology. Three conversational AI models -ChatGPT (OpenAI, San Francisco, CA), Microsoft Bing AI (Microsoft Corp., Redmond, WA), Gemini (Google, Mountain View, CA) - were provided with identical prompts to interpret cases. Their responses were assessed by a panel of five community medicine experts using a 100-point rubric across five domains: diagnosis, intervention, recognition of social determinants, ethical reasoning, and public health appropriateness. Descriptive statistics and Spearman’s rank correlation were used for analysis.

Results

All three AI tools demonstrated near-ceiling performance. Gemini achieved the highest total score (97.00 ± 1.74), excelling in diagnosis and public health appropriateness. ChatGPT (96.00 ± 2.98) performed best in intervention suggestions, while Bing AI (95.70 ± 3.64) showed slightly lower but comparable scores. Correlation analysis revealed weak-to-moderate alignment, with limited statistically significant associations across domains.

Conclusions

ChatGPT, Gemini, and Bing exhibit broadly similar capabilities in interpreting clinico-social cases, with domain-specific strengths. Their complementary nature suggests that they can aid medical education and public health practice, but should supplement rather than replace human expertise to minimize bias and errors.

## Introduction

The integration of artificial intelligence (AI) into healthcare has led to profound and transformative changes in clinical decision-making, patient engagement, and medical education [1,2]. Among these developments, conversational AI models such as ChatGPT (OpenAI, San Francisco, CA), Microsoft Bing AI (Microsoft Corp., Redmond, WA), and Gemini (Google, Mountain View, CA) have emerged as accessible tools capable of processing complex information and providing context-aware responses. Their applications in healthcare are expanding, especially in interpreting case studies, supporting education, and enhancing health communication. By processing extensive medical texts, AI models offer educators and students valuable and relevant guidance.

Community medicine is a field focusing on preventive care, addressing social determinants of health, and implementing interventions at the population level [3]. This speciality constantly involves examining complex clinico-social case studies that integrate clinical information with social, economic, and environmental aspects, necessitating detailed analysis. AI tools capable of processing and interpreting these multifaceted cases can assist medical students, healthcare providers, and public health workers in understanding the complex social factors that influence health outcomes.

While prior research has explored the role of AI in clinical decision support, few studies have systematically evaluated the performance of multiple AI platforms in interpreting socially contextualized case studies in community medicine and public health. This study aimed to compare the performance of ChatGPT, Gemini, and Bing in evaluating and interpreting standardized clinico-social case studies. These conversational AI models were assessed for their accuracy, depth of analysis, sensitivity to social determinants of health, and overall utility in educational and clinical contexts.

## Materials and methods

Study design and ethical considerations

This was a comparative cross-sectional study conducted to evaluate the performance of three most commonly used AI platforms - ChatGPT (GPT-4), Gemini (formerly Bard), and Microsoft Bing AI - in interpreting clinico-social case studies within the field of community medicine. Ethical clearance was not required as there were no human participants involved in the study.

Selection of case studies

Thirty standardized clinico-social case study narratives were prepared, reviewed, and finalized by a panel of five experts holding post-graduate qualifications in community medicine and serving as teaching faculty in various medical institutes. These studies were developed following a uniform and systematic approach (standardized) to provide a realistic case description with a clinical-social context. The narratives of these studies were derived from practical scenarios in community settings. They were formulated as standardized case studies following multiple discussions among subject experts, based on their voluntary participation. These case studies were based on practical reflections gained by the faculty during community and field experiences, encompassing both clinical (medical/health-related) and social (environmental, cultural, behavioral, economic) aspects of a health condition. Therefore, the case studies were indirectly derived from the realistic reflections of actual clinico-social cases that experts have encountered in community settings over time. These 30 cases (Table in Appendices) represented common public health scenarios incorporating both clinical and socio-environmental components (13 cases on various communicable diseases, eight cases of non-communicable diseases, seven cases related to maternal and child health, one case from adolescent health problems, and one clinical case associated with social pathology).

AI tools used and evaluation metrics

Three conversational AI models, namely ChatGPT, Gemini, and Microsoft Bing AI, were used to gather the data for evaluation between July and August 2025. Each AI tool was provided with identical prompts for evaluating diagnosis, social analysis, interventions, and public health appropriateness. The prompt was "Act as a community medicine expert to evaluate the following case study and provide a detailed explanation in clinico-social contexts with ethical implications". The prompt was followed by a case study at a time.

Responses from each AI model (Table in Appendices shows the output of all three AI tools used in the study for two different clinico-social case scenarios) were assessed by the same panel of five community medicine faculty members using a standardized rubric, which includes accuracy of complete and specific medical interpretation/diagnosis (20 points); medical intervention suggested (20 points); recognition of social determinants (20 points); ethical reasoning (20 points) and public health appropriateness (20 points). The scores were not given individually by the experts; instead, the Delphi technique was used to assign scores, which is a more structured method of collecting expert opinions. This method typically involves multiple rounds of questionnaires with controlled feedback to reach consensus [4]. Discussion and consensus were about agreement on the score to be given for each domain of every standardized clinico-social case. The scores were not manipulated; instead, the experts gradually converged to reach a single score. Iterative rounds refine opinions, leading to more stable and reliable scoring compared to a one-time individual score. Final scores reflected the collective, refined wisdom of experts rather than scattered individual opinions. Each case was scored out of 100, and mean scores for each tool were calculated across all 30 case studies.

Statistical analysis

Statistical analysis was performed using IBM SPSS Statistics for Windows (version 27.0; Released 2020; IBM Corp., Armonk, NY). The study employed descriptive statistics to present domain-wise mean scores with standard deviations (SD) for each AI tool in evaluating and interpreting standardized clinico-social case studies. To examine the relationships between the performance of different AI tools across various domains, Spearman’s rank correlation coefficient (r) was calculated for each pairwise comparison of tools within each domain and for total scores. The corresponding p-values were reported to assess statistical significance, with a p-value of less than 0.05 considered statistically significant. Analyses were conducted separately for each domain, and no correlation was computed between scores from the same AI tool.

## Results

In evaluating standardized clinico-social case studies, the three AI tools showed close average scores across domains. ChatGPT scored highest in intervention/treatment suggested (19.60 ± 0.72) and maintained consistent performance across other domains, with a total score of 96.00 ± 2.98. Bing AI had slightly lower scores in some domains, particularly complete and specific medical interpretation/diagnosis (18.80 ± 1.15), resulting in a total score of 95.70 ± 3.64 (Table 1).

**Table 1:** Domain wise scores of ChatGPT, Gemini, and Bing in evaluating and interpreting standardized clinico-social case studies AI: artificial intelligence; SD: standard deviation

Case studies domains	AI tool score, mean ± SD		
	ChatGPT	Bing AI	Gemini
Complete and specific medical interpretation/diagnosis	19.17 ± 0.98	18.80 ± 1.15	19.30 ± 1.14
Intervention/treatment suggested	19.60 ± 0.72	19.57 ± 1.04	19.27 ± 1.38
Recognition of social determinants	19.03 ± 1.35	19.60 ± 0.72	18.93 ± 1.50
Ethical reasoning	19.00 ± 1.93	19.50 ± 1.00	18.93 ± 1.74
Public health appropriateness	19.20 ± 1.44	19.53 ± 1.00	19.27 ± 1.20
Total score	96.00 ± 2.98	95.70 ± 3.64	97.00 ± 1.74

**Figure 1:**
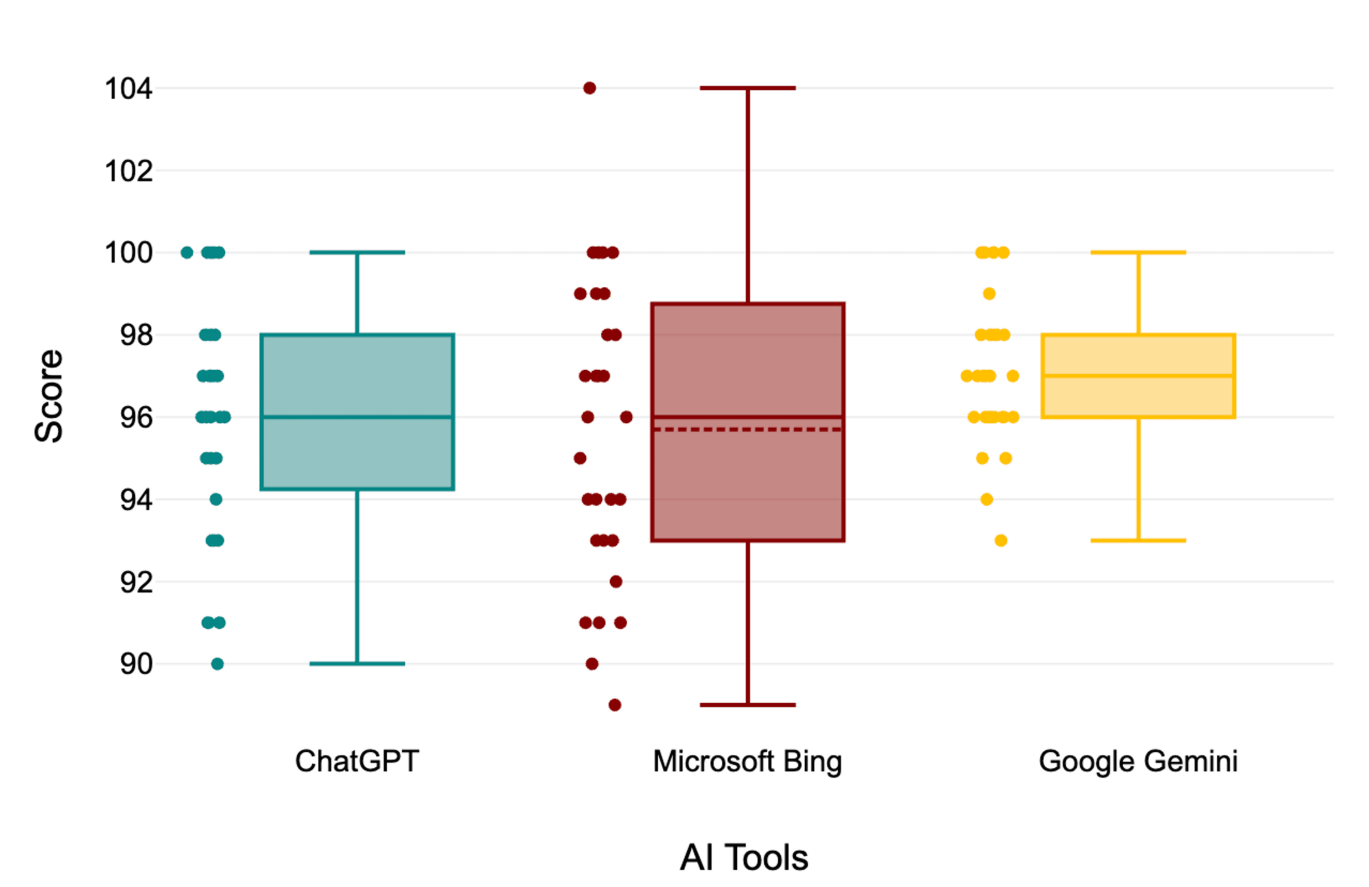
Total scores of ChatGPT, Gemini, and Bing in evaluating and interpreting standardized clinico-social case studies AI: artificial intelligence

Gemini demonstrated the highest total score (97.00 ± 1.74), with particularly strong performance in complete medical interpretation/diagnosis (19.30 ± 1.14) and public health appropriateness (19.27 ± 1.20). Overall, the differences in means between the AI tools were small, suggesting broadly comparable performance with slight domain-specific variations (Figure 1).

Correlation analysis (Table 2) showed mostly weak to moderate relationships between the outputs of the AI tools, with only a few reaching statistical significance (p<0.05). Specifically, ChatGPT and Gemini demonstrated a significant positive correlation in the domain of complete and specific medical interpretation/diagnosis (r=0.36, p=0.04), indicating similar scoring patterns. Additionally, Bing and Gemini had a significant correlation in recognizing social determinants (r=0.38, p=0.03). However, other domains, such as intervention/treatment recommendations, ethical reasoning, and public health relevance, did not show significant correlations between the AI tools. Moreover, total scores across tools were not significantly correlated, suggesting that while some individual domains may align between certain tools, overall evaluation approaches vary among them.

**Table 2:** Correlation between ChatGPT, Gemini, and Bing in evaluating and interpreting standardized clinic-social case studies ^#^r not generated across the same AI tool comparison; ^*^p-value less than 0.05 considered significant AI: artificial intelligence; r: Spearman's correlation coefficient

Case studies domains	AI tool					
	ChatGPT		Bing		Google Gemini	
	r	p-value	r	p-value	r	p-value
Complete and specific medical interpretation/diagnosis						
ChatGPT	NA^#^		0.034	0.85	0.36	0.04
Bing	0.034	0.85	NA^#^		0.33	0.06
Gemini	0.36	0.04^*^	0.33	0.06	NA^#^	
Intervention/treatment suggested						
ChatGPT	NA^#^		-0.24	0.19	0.15	0.41
Bing	-0.24	0.19	NA^#^		0.05	0.77
Gemini	0.15	0.41	0.05	0.77	NA^#^	
Recognition of social determinants						
ChatGPT	NA^#^		-0.21	0.25	0.04	0.80
Bing	-0.21	0.25	NA^#^		0.38	0.03
Gemini	0.04	0.80	0.38	0.03	NA^#^	
Ethical reasoning						
ChatGPT	NA^#^		0.25	0.18	-0.03	0.84
Bing	0.25	0.18	NA^#^		0.04	0.80
Gemini	-0.03	0.84	0.04	0.80	NA^#^	
Public health appropriateness						
ChatGPT	NA^#^		0.22	0.23	-0.08	0.64
Bing	0.22	0.23	NA^#^		-0.13	0.47
Gemini	-0.08	0.64	-0.13	0.47	NA^#^	
Total score						
ChatGPT	NA^#^		0.102	0.591	-0.006	0.97
Bing	0.102	0.591	NA^#^		0.31	0.09
Gemini	-0.006	0.97	0.31	0.09	NA^#^	

## Discussion

The majority of studies conducted so far have primarily explored the utility of three widely accessible large language model (LLM)-based AI tools - ChatGPT, Bing AI, and Google Gemini - within clinical, investigative, or radiological domains. These investigations have generally emphasized the diagnostic accuracy, interpretative capacity, and reasoning consistency of such models in structured medical care settings. By contrast, the present study is the first to examine the application of these AI tools in interpreting community-based clinico-social case studies, thereby extending the scope of inquiry beyond the hospital or laboratory setting into the broader field of community medicine and primary health care.

Our findings demonstrated that all three AI models yielded mean scores approaching ceiling levels across multiple evaluative domains, including diagnosis, intervention planning, ethical reasoning, assessment of social determinants of health, and public health perspectives. While Gemini marginally outperformed the others by achieving the highest total score, ChatGPT and Bing exhibited narrowly lower yet comparable results, reflecting a relatively balanced performance across the models. The differences observed suggest that although Gemini may hold a slight edge in diagnostic precision, ChatGPT maintains distinct strengths in intervention-related reasoning, with Bing performing consistently but without a clear leading advantage.

These results align with prior domain-specific investigations. For example, earlier evaluations in clinical neuro-ophthalmology showed ChatGPT achieving correct diagnoses in 40% of cases, a performance level similar to Bing and Gemini, but with greater diagnostic suitability (60% compared with 50% for the others) [5]. These findings align with our observation that Gemini excels in diagnostic interpretation, while ChatGPT demonstrates strong utility in guiding interventions. In thoracic radiology, ChatGPT 3.5 was reported to outperform both human radiologists and other AI models, with a diagnostic accuracy of 53.2%, underscoring its ability to reason through complex medical imaging data [6]. Similarly, a recent meta-analysis synthesizing results across diverse medical queries found ChatGPT to achieve an overall accuracy of approximately 56% [7], while another study evaluating GPT-4 demonstrated a correct diagnosis rate of 57%, surpassing 99.98% of simulated human readers generated from online responses [8].

Collectively, the evidence suggests that ChatGPT, across its various iterations, has consistently emerged as a highly competitive model, often outperforming comparators such as Bing and Gemini in accuracy when applied to structured medical vignettes [9-12]. Nonetheless, some studies caution against uncritical reliance, emphasizing that while ChatGPT may contribute value in clinical reasoning exercises, its outputs require careful human oversight and verification to mitigate potential errors and biases [13]. A study conducted to compare the performance of these three LLMs with each other highlighted their potential as supportive tools in medical education and clinical practice, especially when tailored to specific fields [14]. Another study conducted to compare the diagnostic capability of these three LLMs in challenging clinical cases indicated that LLMs produced varying responses to identical clinical input, with Gemini performing the strongest [15]. Our study found that both ChatGPT and Gemini performed slightly better than Bing, a finding that has been consistently observed across other clinical scenarios, such as surgical decision-making and dentistry [16-18].

This comparative analysis demonstrates that current AI tools have achieved broadly comparable competence in clinical-social case evaluation, while maintaining distinct analytical characteristics. The finding that similar overall performance can be achieved through different domain-specific strengths suggests a maturation of AI clinical reasoning capabilities. However, the variation in correlations across domains indicates that these tools are not merely different interfaces to identical reasoning systems, but instead represent genuinely different approaches to clinical analysis. This diversity may ultimately prove beneficial for clinical practice, offering healthcare providers multiple AI perspectives to inform patient care decisions in field settings.

Limitations

The evaluation of AI tools was based on standardized clinical cases, which, while applicable for comparison, do not fully reflect the complexity, variability, and unpredictability of real-world clinical practice. In actual healthcare settings, patients often present with vague, overlapping, or atypical symptoms, and diagnoses may require integrating contextual, emotional, or non-verbal cues-factors that standardized cases do not account for. As such, the performance patterns observed in this controlled evaluation may not directly translate to real clinical environments, particularly in cases involving rare diseases, novel presentations, or situations that require nuanced clinical judgment and intuition. Additionally, this evaluation represents a snapshot in time. The AI tools assessed are regularly updated, and their capabilities can change significantly with future iterations. Therefore, the findings here may not reflect their long-term performance or their potential after improvements. It is also important to note that this evaluation was conducted using free-to-use versions of the tools. Premium or enterprise versions often come with enhanced features, access to more advanced models, or integration with external medical databases, which may yield more accurate or contextually rich responses. The results may differ between free-to-use and premium versions of the LLMs.

## Conclusions

ChatGPT, Gemini, and Bing AI demonstrated comparable abilities in interpreting clinico-social cases, though each shows unique domain-specific strengths. Their complementary features highlight potential applications in medical education and public health, yet they must function as supportive tools rather than replacements for human expertise to prevent bias and minimize errors. Our study highlights that while all three models perform effectively, none excels across every domain, echoing findings from earlier research. These results emphasize that AI integration in healthcare requires caution, with consistent involvement of human professionals to ensure accuracy, reduce risks of misinterpretation, and maintain ethical responsibility.

## Appendices

**Table 3:** The clinico-social case scenarios used in the study

Case study	Case description	Category
1	A 10-year-old girl and her 4-year-old brother presented with severe itching, particularly worse at night, affecting multiple body areas. Physical examination revealed superficial black, tortuous tunnels along with small red papules and blisters in the interdigital spaces.	Communicable disease
2	A 65-year-old farmer has three sons who, along with their wives and children, lived with him in a joint family for several years. Following personal disputes, the sons separated, divided the property and land without their father’s consent, and refused to take responsibility for his care. As an elderly citizen of India, what legal rights and protective laws are available to safeguard this farmer?	Social pathology
3	A 10-year-old boy, while playing in his village streets, was bitten by a stray dog that had already bitten 8–9 individuals in the past week. He presented to the Community Health Centre (CHC) with multiple dog bite wounds on his left leg.	Communicable disease
4	A 34-year-old man presented with complaints of weight loss, evening fever, and cough. He reported similar symptoms the previous year and had received treatment. On advice from the village health worker, he visited the nearby primary health care centre. The medical officer elicited that the patient had taken anti-tuberculosis drugs for only three months before stopping when he felt better. Clinical examination was followed by sputum microscopy, which showed confirmed acid-fast bacilli. Cartridge-Based Nucleic Acid Amplification Test (CBNAAT) showed rifampicin resistance.	Communicable disease
5	A 17-year-old student reported fatigue and occasional dizziness. She had no significant past history and did not consume alcohol or tobacco. Her parents were healthy. On examination, she had pale skin, conjunctiva, and nail beds, but her cardiovascular system was normal. Vitals: BP 115/75 mmHg, Pulse 90/min, BMI 19.5 kg/m², with onsite hemoglobin estimation reported at 7.2 g/dL.	Adolescent health problems
6	A 52-year-old man presented with itching and irritation in the groin region, along with a rash. He had no previous dermatological history. Examination revealed a reddish-brown rash with a well-demarcated edge involving the upper thighs and groin, extending to the scrotum. No rash was present elsewhere. Identify the condition with differential diagnoses and outline management.	Communicable disease
7	A 52-year-old homemaker was referred by an ANM after her blood pressure was found to be 162/94 mmHg during screening. She had no symptoms. Anthropometry: Weight 62 kg, Height 156 cm, Waist circumference 78 cm.Habits: No tobacco or alcohol use.	Non-communicable disease
8	Mr. A sought advice regarding persistently high blood pressure over the past year. He had previously been prescribed medication but avoided taking it, fearing dependence. Today’s blood pressure (BP): 156/100 mmHg. Anthropometry: Weight 82 kg, Height 168 cm, Abdominal circumference 98 cm. Habits: No tobacco or alcohol use.	Non-communicable disease
9	A 48-year-old farmer was referred after his fasting blood glucose at a sub-centre was 188 mg/dL. At the PHC, his fasting plasma glucose was 178 mg/dL. He complained of persistent fatigue. History: Smoked beedis since age 20. Today’s BP: 160/90 mmHg. Anthropometry: Weight 72 kg, Height 168 cm.	Non-communicable disease
10	A 55-year-old male presented with fever and cough with expectoration lasting more than two weeks. He had been treated with antibiotics and cough syrup for one week without improvement. At the PHC, investigations confirmed tuberculosis. He lives with his two grandchildren—one newborn and one 2-year-old—in the same house.	Communicable disease
11	A 22-year-old female delivered a baby girl weighing 2.2 kg at the PHC today. She is scheduled for discharge within two days. She has a 3-year-old son who is fully immunized. Her husband, a factory supervisor, earns ₹8000 per month and is a chronic smoker.	Maternal and child health
12	A 28-year-old housewife presented with 2-month amenorrhea. She has three children aged 6, 4, and 2 years. Her husband, a 32-year-old auto-rickshaw driver, earns ₹6000 per month. They live in a rented two-room house and consume well water. None of the children were immunized, and they suffer frequent diarrheal episodes. She reported that her husband regularly uses condoms for family planning, yet she now experiences amenorrhea.	Maternal and child health
13	A 28-year-old postman was bitten on the leg by a dog while delivering mail in a village. The bite caused bleeding, and he was rushed to the PHC within 2 hours of the incident.	Communicable disease
14	A 24-year-old housewife presented at the PHC with her four children aged 8, 5, 4, and 2 years. All appeared malnourished. The youngest weighed only 8 kg, and both under-five children were unimmunized. Her husband is a daily wage laborer with alcohol dependence.	Maternal and child health
15	A 51-year-old electrician presented with weakness of the right upper and lower limbs for two days. He had had hypertension for five years but was not on medication. Two years ago, he discontinued antihypertensive drugs after a homeopath declared his BP “normal.” Anthropometry: Height 170 cm, Weight 70 kg	Non-communicable disease
16	A 28-year-old woman with 25 weeks of amenorrhea attended the medical college hospital for antenatal care. On examination, she was normal, but she tested HIV-positive. Her husband, a factory worker in Mumbai, visits her twice yearly. She lives with her in-laws and two children aged 5 years and 2.5 years.	Maternal and child health
17	A 2-year-old girl presented with loose stools for two days. She was alert but restless. She is the youngest of four siblings. Her parents are daily wage workers who live with her paternal grandparents in a Rae Bareli colony. The elder siblings are aged 4 to 8 years.	Communicable disease
18	A 50-year-old widow reported fever and burning micturition for three days. Findings: BMI 31 kg/m^2^, Hemoglobin 10 g/dL, BP 130/80 mmHg, pus cells ++ in urine, FBS 120 mg/dL, PPBS 300 mg/dL. History: Mother had diabetes for 18 years before dying of a heart attack. Her husband, a driver, died 4 months ago due to a head injury from a road accident. She now works part-time as a domestic helper, earning ₹5000/month, and supports two daughters aged 21 and 23 years.	Communicable disease
19	Mr. X, a 65-year-old widower, was admitted with left-sided paralysis. He had hypertension for 15 years and smoked heavily for 40 years. On Examination: BP 150/100 mmHg, BMI 31 kg/m^2^.	Non-communicable disease
20	A 3-year-old girl was brought in with high-grade fever and rash lasting one week. Rash appeared four days after fever onset. She also had a cough, a congested conjunctiva, and an inflamed throat. Koplik spots were seen in the upper buccal mucosa. The child, from a tribal village, weighed 11 kg. Her illiterate mother reported frequent respiratory and diarrheal illnesses in her children. This was her fourth pregnancy.	Communicable disease
21	Mr. X, a 45-year-old rubber plantation owner, was admitted with seven days of high fever, severe body ache, joint pain, retro-orbital pain, and gastrointestinal symptoms. On Examination: Temperature 102°F, rashes on face/neck/chest, purpuric spots in oral cavity. History: A similar febrile illness with rashes two years ago was treated at a medical store. Lives with wife, three children, and a maid in the PHC catchment area.	Communicable disease
22	A 32-year-old construction laborer from Lalganj, presented with four days of fever, chills, rigor, and sweating. He reported fever episodes on alternate days. Peripheral smear for parasites was ordered. He lives in a labor camp with his pregnant wife and other workers in poor sanitary conditions with stagnant water and open tanks.	Communicable disease
23	A 23-year-old woman delivered a full-term baby weighing 2 kg at the medical college hospital. She was discharged on the third postnatal day. On the fifth day, during a home visit, the MPW noted the mother was febrile with left breast engorgement, while the baby was breastfeeding well. This was her fourth pregnancy (one abortion). She has two daughters (aged 3 years and 18 months). Her husband, a taxi driver, lives with his parents in a three-room house.	Maternal and child health
24	Mr. X, a 56-year-old widower, presented with headache, giddiness, palpitations, breathlessness on exertion, and bilateral leg swelling. He was diagnosed with hypertension five years earlier, but discontinued treatment due to economic hardship. He quit his job two years ago and now depends on his son’s family. He smoked and consumed alcohol for 20 years. On Examination: Pedal oedema, BP 146/98 mmHg, Body mass index (BMI) 31 Kg/m^2^, mild cardiomegaly.	Non-communicable disease
25	A 25-year-old woman attended an antenatal check-up at 20 weeks of gestation. She had registered at 12 weeks and visited again at 16 weeks. She has two children (both caesarean section) and a history of three consecutive first-trimester abortions. Dietary history revealed protein deficiency. Her husband works as a shop helper, and the family belongs to socioeconomic class IV. Findings: Hemoglobin 10 g/dL, BP 130/90 mmHg, no obstetric abnormalities.	Maternal and child health
26	A 54-year-old business executive presented with a non-healing ulcer on the left foot for one month. He had type II diabetes for four years and was on insulin for two years. Due to work commitments, he was irregular with consultations and treatment. He noted fluctuating sugar levels on occasional private testing. He had gained weight recently. History: One year ago, admitted with sudden loss of consciousness. Father and elder brother also have diabetes. On Examination: BMI 32 Kg/m^2^	Non-communicable disease
27	A 3-year-old girl was admitted with fever, cough, and breathlessness for two days. Five days earlier, she had a runny nose, sneezing, and fever, followed by rashes spreading over her body. She later developed a severe cough and breathlessness. History: Immunized only with Bacillus Calmette-Guérin (BCG), Diphtheria-Pertussis-Tetanus (DPT), and oral polio vaccine (OPV). Registered at Anganwadi but irregular due to parents’ construction work.	Communicable disease
28	Mr. A, a 70-year-old widower, was admitted with left-sided paralysis. He also complained of exertional dyspnea. He had hypertension for 15 years, but was irregular with treatment due to financial constraints. He was a chain smoker for 40 years. On Examination: BP 150/100 mmHg, BMI 32, pedal edema, cardiomegaly on X-ray. History: Lives with son, daughter-in-law, and three grandchildren.	Non-communicable disease
29	A 35-year-old primigravida at 34 weeks was admitted with pregnancy complications. She had a prior admission at 20 weeks for vaginal bleeding. She later attended only one antenatal clinic visit, receiving tetanus toxoid and one month of iron-folic acid supplementation. She works as a part-time housemaid, performing strenuous household tasks for four families. Family: Lives in a joint family with husband, in-laws, and children of brother-in-law. On Examination: Height 126 cm, BP 140/100, mmHg, Hemoglobin 10 g/dL, Cephalic presentation, fetal heart rate was 110/min, pedal edema +.	Maternal and child health
30	Mr. A, a 40-year-old farmer involved in paddy cultivation, presented with five days of fever, myalgia, conjunctival suffusion, abdominal pain, jaundice, and reduced urine output. A rise in fever cases was noted over the past month following heavy rainfall lasting five days. On Examination: Facial puffiness and palpable tender liver were present.	Communicable disease

**Table 4:** Output generated by all three LLMs for two different clinico-social case scenarios LLMs: large language models

Case description	ChatGPT	Google Gemini	Microsoft Bing
A 10-year-old girl and her 4-year-old brother presented with severe itching, particularly worse at night, affecting multiple body areas. Physical examination revealed superficial black, tortuous tunnels along with small red papules and blisters in the interdigital spaces.	Diagnosis Clinical clues:10-year-old girl and 4-year-old brother, severe itching, especially worse at night, involvement of interdigital areas (spaces between fingers), superficial black tortuous tunnels, small red papules, or blisters. Most likely diagnosis: Scabies (Sarcoptes scabiei infestation) Rationale: Classic symptoms include intense pruritus (itching) worse at night. Physical findings such as burrows (“black tortuous tunnels”), papules, and vesicles, especially in interdigital spaces, are characteristic, spread is often among close contacts, suggesting both children have acquired it from a shared source (likely home or school). Social analysis Key social aspects: Age group: young children – vulnerable population, shared condition: likely common source or living condition (e.g., overcrowding, poor hygiene), family/household dynamics: possible lack of awareness or resources for hygiene/sanitation, environmental factors: possible poverty, limited access to healthcare, poor water availability, or shared bedding Implications: The infestation likely reflects broader social determinants like: low socioeconomic status, overcrowded living conditions, limited access to preventive care, health illiteracy in caregivers, school exposure might be a source or a risk for further spread, raising public health concerns. Intervention Clinical intervention: Prescribe topical permethrin 5% cream (first-line treatment), consider oral ivermectin if topical is not feasible, treat all household members, even if asymptomatic, wash clothing, bedding, and towels in hot water, educate about preventing reinfection Social intervention: Home visit or social worker referral (if indicated) to assess living conditions, health education: hygiene, laundry care, identifying symptoms, school-based health check-up or awareness campaign, referral to community health programs or NGOs if poor living conditions or neglect is suspected Ethical reasoning Principles involved: Beneficence: Prompt treatment to relieve suffering, non-maleficence: avoiding harm by ensuring all infected individuals are treated to prevent reinfestation, justice: ensuring access to treatment regardless of socioeconomic status, autonomy: informed consent from caregivers about treatment plan and public health implications Ethical concerns: Stigma: Families may be blamed or shamed for poor hygiene, confidentiality: especially in school/community settings—must balance individual privacy with public health responsibilities, Neglect: If signs suggest child neglect (e.g., chronic skin conditions left untreated), mandatory reporting may be ethically and legally required	Medical diagnosis: Based on the symptoms described (severe itching, worse at night, affecting different body areas, and the presence of "superficial black tortuous tunnels and small red papules or blisters in the interdigital areas"), the medical diagnosis is highly suggestive of scabies. Rationale: The "black tortuous tunnels" are characteristic burrows created by the Sarcoptes scabiei mite, and the intense nocturnal itching, along with papules and blisters, are classic signs of a scabies infestation. The involvement of interdigital areas is also a common site for scabies. Social/Familial Diagnosis (Initial thoughts): While not a formal "diagnosis" in the medical sense, the presentation of two young siblings from the same household with an infectious skin condition immediately raises concerns about: Household transmission: Scabies is highly contagious and spreads through prolonged skin-to-skin contact. It's highly probable that other household members are also infected or at risk. Socioeconomic factors: While scabies can affect anyone, poor hygiene, overcrowding, and limited access to healthcare are risk factors that might be present and warrant exploration. Impact on children: The severe itching can significantly disrupt sleep, concentration, and overall well-being, affecting school performance and social interactions. Social analysis: This is where a social worker's perspective becomes crucial. Family System: Who are the primary caregivers? Are parents present and engaged? What is the family structure like (single-parent, two-parent, extended family)? Are there other children in the household? What is their health status? What is the family's understanding of health and hygiene? Environmental factors: Housing conditions: Is the home overcrowded? Are living conditions clean and conducive to preventing infestations? Access to resources: Does the family have access to clean water, laundry facilities, and consistent bathing supplies? Community context: Are there other cases of scabies in the community or school, suggesting a broader public health issue? Socioeconomic factors: Poverty: Limited financial resources can impact access to medication, proper clothing, laundry facilities, and even adequate nutrition, which can affect immune response. Employment: Are parents employed? Does their work schedule allow for adequate caregiving and healthcare appointments? Education level: The parents' understanding of health information and ability to follow treatment protocols might be influenced by their education. Cultural factors: Are there any cultural beliefs or practices that might influence health-seeking behaviors or adherence to treatment? Child's Functioning: School attendance/Performance: Is the severe itching affecting their ability to attend school or concentrate on studies? Social isolation/Stigma: Are the children experiencing any social isolation or bullying due to their condition? Emotional Well-being: How are the children coping emotionally with the discomfort and potential embarrassment? Intervention Intervention should be multi-faceted, addressing both the immediate medical needs and the underlying social determinants of health. Medical intervention (Collaboration with dermatology clinic): Treatment adherence: Ensure parents understand the prescribed scabicide treatment (e.g., permethrin cream) and how to apply it correctly to all affected individuals in the household, not just the symptomatic children. Environmental decontamination: Educate the family on the importance of washing all clothing, bedding, and towels in hot water and drying on a hot cycle, or sealing non-washable items in plastic bags for at least 72 hours. Follow-up: Schedule follow-up appointments to monitor treatment effectiveness and ensure complete eradication. Social work intervention: Psychoeducation: Provide clear, non-judgmental information about scabies, its transmission, and the importance of treating the entire household. Address any misconceptions or shame. Resource connection: Financial assistance: If poverty is a factor, connect the family to programs for medication assistance, laundry facilities, or housing support. Hygiene supplies: Help access soap, shampoo, and clean clothing if needed. School liaison: Communicate with the school (with parental consent) to address any attendance issues or potential bullying and to educate school staff about the condition if necessary. Public health: If a community outbreak is suspected, collaborate with local public health agencies. Family support: Offer emotional support to the parents and children, help parents develop strategies for managing the itching and discomfort. Advocacy: Advocate for the family's needs within the healthcare system or with other community agencies. Home visit (if appropriate and consented to): A home visit could provide invaluable insight into living conditions and help assess the feasibility of environmental decontamination. Safety planning (if applicable): While not immediately apparent, if neglect is identified as a contributing factor, a safety assessment and potential report to child protective services would be warranted. Ethical reasoning: Several ethical principles guide the social worker's approach in this case: Beneficence: The primary goal is to do good and promote the well-being of the children and their family. This means ensuring effective treatment and addressing underlying factors contributing to their suffering, non-maleficence: Avoid doing harm. This includes not blaming or shaming the family for the condition, and ensuring interventions do not inadvertently create new problems, autonomy/ self-determination: Respect the family's right to make decisions about their care, provide information in an understandable way so they can make informed choices, while advocating for treatment, recognize their right to decline, and then explore the reasons behind such decisions, justice: Ensure equitable access to resources and treatment, regardless of socioeconomic status or background, challenge any systemic barriers that might prevent the family from receiving adequate care, confidentiality: Maintain the privacy of the family's health information, sharing only what is necessary with other professionals with informed consent, dignity and worth of the person: Approach the family with respect and empathy, recognizing their inherent worth regardless of their circumstances or the challenges they face, avoid judgmental attitudes regarding hygiene or living conditions, competence: practice within the scope of one's professional competence, collaborating with medical professionals for the accurate diagnosis and treatment of the physical condition. Ethical dilemmas/ considerations: Balancing confidentiality with child protection: If the social worker suspects child neglect (e.g., repeated untreated infestations, lack of basic hygiene), there's a duty to report to child protective services, which overrides confidentiality. This would require careful assessment and documentation. Cultural sensitivity vs. Best practice: If cultural beliefs conflict with recommended medical interventions, the social worker must navigate this sensitively, aiming for culturally competent care while still prioritizing the children's health. Resource scarcity: In situations of limited resources, how does the social worker prioritize needs and advocate for the family without overpromising or creating unrealistic expectations? Stigma: How to help the family cope with the potential social stigma associated with scabies without minimizing the medical seriousness of the condition.	Diagnosis Clinical presentation: Symptoms: Severe nocturnal itching, superficial black tortuous tunnels, red papules/blisters, location: Interdigital areas (between fingers), common site for infestation. Likely Diagnosis: Scabies (Sarcoptes scabiei infestation): Caused by a parasitic mite that burrows into the skin, classic signs include intense itching (especially at night), burrows, and papular rash, highly contagious, especially in close-contact environments like households or schools. Social analysis Family and environmental context: Age of children: 10 and 4 years old—both vulnerable and dependent on caregivers. Possible contributing factors: Overcrowded living conditions: Scabies spreads rapidly in crowded homes, poor hygiene or limited access to clean water, neglect or lack of parental supervision, school or daycare exposure. Social Concerns: Stigma and isolation: Children may be excluded from school or social activities. Parental capacity: Are caregivers aware and capable of managing hygiene and treatment? Economic hardship: May limit access to medical care, clean clothing, or proper treatment. Intervention Medical treatment: Topical scabicides: Permethrin cream or oral ivermectin, treat all household members simultaneously, environmental decontamination: Washing bedding, clothing, and towels in hot water. Social Work Interventions: Home visit: Assess living conditions, hygiene practices, and caregiver capacity. Education: Teach family about transmission, treatment, and prevention. Referral: Link to community health services or public assistance if needed. School coordination: Ensure children are not unfairly excluded and receive support. Ethical reasoning Key ethical considerations: Confidentiality: Protect the family's privacy, especially in school or community settings. Non-judgmental approach: Avoid blaming caregivers; focus on support and education. Child welfare: If neglect or abuse is suspected, mandatory reporting may be required. Equity: Ensure access to treatment regardless of socioeconomic status. Balancing rights: Children’s right to health and safety vs. family autonomy and dignity. Public health concerns vs. individual confidentiality.
A 65-year-old farmer has three sons who, along with their wives and children, lived with him in a joint family for several years. Following personal disputes, the sons separated, divided the property and land without their father’s consent, and refused to take responsibility for his care. As an elderly citizen of India, what legal rights and protective laws are available to safeguard this farmer?	Diagnosis Primary Concern: Elder neglect and abandonment Symptoms/Indicators: Loss of family support, possible emotional distress, isolation, and financial insecurity, risk of depression, malnutrition, homelessness, or physical health deterioration Probable diagnosis: Psychosocial elder abuse: includes emotional neglect and abandonment, possible adjustment disorder or depressive disorder, if clinically assessed, may be vulnerable to exploitation or homelessness. Social analysis Family structure: Traditional joint family system disrupted, breakdown of intergenerational caregiving, patriarch (father) was not consulted or cared for. Key social factors: Erosion of traditional values, lack of legal awareness in rural areas, ageism: older adults seen as a burden, economic dependence on children, lack of pension, social security, or health insurance Intervention Immediate interventions: Medical & psychological assessment for stress, depression, and physical health, referral to social worker or community welfare officer Community & institutional support: Link with senior citizen welfare programs (e.g., pensions, health schemes), Shelter or old age home referral if necessary, NGO or District Legal Services Authority (DLSA) for legal aid Ethical & Legal Reasoning Legal Rights of Elderly in India: Maintenance and welfare of Parents and Senior Citizens Act, 2007, legally obligates children and heirs to maintain parents (biological, adoptive, or step-parents), elderly person can claim monthly maintenance up to ₹10,000, can approach Maintenance Tribunal for relief, provision for speedy redressal of grievances (within 90 days). Section 125 of Criminal Procedure Code (CrPC): Entitles parents to claim maintenance from sons/daughters if they are unable to maintain themselves. Article 41 of the Constitution of India (Directive principles) Ensures the right to public assistance in old age and in cases of undeserved want. Senior citizen helplines and schemes Example: Elderline (14567) – National helpline for elder abuse Indira Gandhi National Old Age Pension Scheme (IGNOAPS) State-specific welfare schemes Ethical Considerations: Principle application in this case, Justice: equal access to care and legal recourse for elderly regardless of family status, Autonomy: The father’s consent was ignored in division of property, Beneficence : Systems must act to ensure his well-being Non-maleficence: Prevent further harm from isolation or poverty Dignity: Uphold the father's dignity despite familial rejection	Diagnosis Social diagnosis (primary): Elder neglect and abandonment. Manifestations: Physical neglect: Implied, as no one is ready to take responsibility for his care, which would include food, shelter, and medical needs. Emotional/psychological neglect: The father has been cast aside by his own children, leading to immense emotional distress, feelings of betrayal, loneliness, and loss of dignity. Financial exploitation/deprivation: The most explicit form of abuse here. The sons have illegally distributed the father's property without his consent, leaving him without his primary means of livelihood and shelter. Medical/Physical health (inferred): A 65-year-old farmer, likely with a history of physical labour, is at risk of age-related health issues (e.g. joint pain, chronic diseases), without care, his health could rapidly deteriorate due to lack of food, shelter, and medical attention. Mental health (inferred): High risk of depression, anxiety, grief, and feelings of helplessness due to the sudden and devastating loss of family support, home, and financial security. Social analysis This situation highlights a breakdown in traditional Indian family values and responsibilities, compounded by legal and economic factors. Family dynamics: Breakdown of joint family system: The case explicitly describes the dissolution of a joint family due to "personal disputes," indicating internal strife and lack of cohesion. Filial irresponsibility: The core issue is the complete abandonment of the father by his sons, directly contradicting societal and legal expectations of filial piety and care for elderly parents. Lack of consensus and respect: The distribution of property without the father's consent demonstrates a severe disrespect for his authority, legal rights, and well-being. Gender roles (implicit): While the sons are primarily responsible, the mention of "wives and children" getting separated suggests a complex dynamic where wives might also influence the decision-making or be unable/unwilling to provide care. Socioeconomic context: Vulnerability of elderly farmers: Farmers often rely on their land and children for old-age security. Without this, they are extremely vulnerable, especially if they have limited savings or pension. Property as security: For many in rural India, land and home are their only assets. Losing these can be catastrophic. Changing economic landscape: Economic aspirations and individualistic tendencies among younger generations can sometimes lead to neglect of traditional responsibilities. Cultural context: Erosion of traditional values: Indian culture traditionally places a high value on respecting and caring for elders, this case represents a severe deviation from these norms, indicating a breakdown of social fabric at the family level. Stigma: The farmer might face significant social stigma within his community due to being abandoned, potentially leading to further isolation. Power imbalance: The sons, being younger and perhaps more financially capable (or at least having taken control of the assets), wield significant power over their elderly father, who is now dispossessed and dependent. Intervention: Interventions would need to be immediate, legal, and social, focusing on the farmer's safety, maintenance, and dignity. Immediate needs: Shelter and food: Secure immediate temporary shelter (e.g., a relative's home, local old age home, or community support), ensure access to food and basic necessities. Medical assessment: A comprehensive medical check-up to assess his physical health status and address any immediate concerns. Emotional support: Provide counselling and emotional support to help him cope with the trauma of abandonment and loss, legal intervention (Crucial and primary): Maintenance claim: The farmer must be immediately informed of and assisted in filing a claim for maintenance from his sons under the Maintenance and Welfare of Parents and Senior Citizens Act, 2007. This act makes it a legal obligation for children to maintain their parents, an application can be filed with the Maintenance Tribunal (constituted under the Act), The Tribunal can order the sons to pay a monthly allowance, The process is designed to be inexpensive and speedy (aiming for disposal within 90 days), Non-compliance can lead to imprisonment. Property Reclamation: Under Section 23 of the Maintenance and Welfare of Parents and Senior Citizens Act, 2007, if a senior citizen has transferred property (like a house or land) to a relative (including children) with the condition that they would provide basic amenities and physical needs, and the relative neglects or refuses to provide such maintenance, the transfer of property can be declared void by the Tribunal. This is a critical provision for the farmer to regain his land and house. If the property was distributed without any formal transfer document from the father, it's an illegal occupation, and a civil suit for possession might be necessary, alongside police assistance if there's resistance. Criminal Procedure Code (CrPC) Section 125: The farmer can also seek maintenance under Section 125 of the CrPC, 1973. While the Maintenance and Welfare of Parents and Senior Citizens Act, 2007 is more specific and comprehensive for senior citizens, CrPC 125 also provides for maintenance to parents unable to maintain themselves. It's important to note that a parent cannot claim maintenance under both acts simultaneously. The social worker should help the farmer choose the most effective legal path. Social Support and Advocacy: Community mobilization: Engage local community leaders, village elders, and panchayat members to mediate and pressure the sons to fulfill their responsibilities, social pressure can sometimes be more effective than legal action in rural settings. NGO/Social organizations: Connect the farmer with NGOs working for elder welfare and rights. They can provide legal aid, counselling, and support. Police assistance: If there is any threat or refusal to vacate the property, police intervention may be required to enforce legal orders. Long-term care planning: Depending on the legal outcomes, explore options for stable housing, financial management, and ongoing healthcare. Ethical reasoning: Beneficence and Non-maleficence: The primary ethical duty is to act in the best interest of the elderly farmer (beneficence) and to prevent further harm (non-maleficence). This involves ensuring his physical safety, financial security, and emotional well-being. Justice: The social worker must ensure that the farmer receives justice against the wrongful actions of his sons. This includes upholding his legal rights to property and maintenance and advocating for equitable treatment. Dignity and worth of the person: The farmer's dignity has been severely compromised, interventions must be respectful, empowering, and aimed at restoring his sense of self-worth and autonomy. He should be actively involved in decision-making regarding his future. Self-determination (with caveats): While promoting self-determination, the social worker must also consider the farmer's vulnerability and potential emotional distress which might affect his ability to make fully informed decisions initially. Providing clear information about his rights and options is key. Professional boundaries and collaboration: The social worker needs to collaborate closely with legal professionals (lawyers, legal aid), local authorities, and potentially other social welfare agencies. The social worker's role is not to replace legal counsel but to facilitate access to it and provide holistic support. Confidentiality: While sensitive to the family's privacy, the urgency of elder neglect and the legal implications mean that information may need to be shared with relevant legal and protective services, with the farmer's informed consent where possible, or as legally mandated in cases of abuse. Conflict of interest: The social worker's sole allegiance is to the well-being of the elderly farmer, even if it means confronting the actions of his sons. This case is a stark reminder of the challenges faced by elderly individuals in India when traditional support systems fail and underscores the critical role of social workers in advocating for their rights and well-being through legal and social avenues.	Diagnosis Presenting issue: A 65-year-old farmer has been abandoned by his three sons after a property dispute, the sons divided the property without his consent and refused to take responsibility for his care. Core Problem: Elder neglect and loss of housing and financial security, emotional trauma due to family breakdown and isolation. Social analysis Family dynamics: Breakdown of the joint family system, which traditionally supports elders, disregard for the father's authority and rights in property decisions, possible erosion of cultural values around elder care. Socioeconomic Factors: The farmer may lack independent income, social support, or legal literacy, rural setting may limit access to institutional support or legal recourse. Social consequences: Risk of homelessness, poverty, and mental health decline, Social isolation and loss of dignity Intervention Social Work Actions: Immediate support: Connect the farmer to local welfare schemes, old age homes, or NGOs. Legal aid: Help him file a complaint under relevant laws. Counselling and advocacy: Support emotional well-being and assert his rights. Community engagement: Mobilize local panchayat or community leaders to mediate or support, raise awareness about elder rights and responsibilities of adult children. Ethical reasoning Ethical principles: Justice: The farmer deserves fair treatment and restitution. Autonomy: His consent in property matters must be respected. Beneficence: Actions should promote his well-being. Non-maleficence: Prevent further harm from neglect or exploitation. Dilemma: Balancing family autonomy with the moral and legal duty to care for elders. Legal Rights and Protections in India: The farmer has strong legal protections under Indian law: Maintenance and Welfare of Parents and Senior Citizens Act, 2007 Children are legally obligated to provide maintenance to parents who cannot support themselves, The farmer can file a case before the Maintenance Tribunal to claim monthly financial support, The Act allows for speedy resolution and does not require a lawyer. Constitutional Provisions Article 41: Directs the state to provide public assistance in old age. Article 46: Protects the economic and social interests of weaker sections, 1including elders. Property Rights If the property was ancestral or jointly held, the farmer may have a legal claim to it, unauthorized division without his consent may be challenged in civil court. Welfare Schemes Access to old age pensions, healthcare subsidies, and shelter homes, NGOs and government programs offer support for abandoned or neglected elders.

## References

[1] Bajwa J, Munir U, Nori A, Williams B (2021). Artificial intelligence in healthcare: transforming the practice of medicine. Future Healthc J.

[2] Alowais SA, Alghamdi SS, Alsuhebany N (2023). Revolutionizing healthcare: the role of artificial intelligence in clinical practice. BMC Med Educ.

[3] Shukla R, Mishra AK, Banerjee N, Verma A (2024). The comparison of ChatGPT 3.5, Microsoft Bing, and Google Gemini for diagnosing cases of neuro-ophthalmology. Cureus.

[4] Nasa P, Jain R, Juneja D (2021). Delphi methodology in healthcare research: how to decide its appropriateness. World J Methodol.

[5] Gunes YC, Cesur T (2024). A comparative study: diagnostic performance of ChatGPT 3.5, Google Bard, Microsoft Bing, and radiologists in thoracic radiology cases. medRxiv.

[6] Institute of Medicine (US) Committee on Assuring the Health of the Public in the 21st Century (2003). Understanding population health and its determinants. The Future of the Public's Health in the 21st Century.

[7] Wei Q, Yao Z, Cui Y, Wei B, Jin Z, Xu X (2024). Evaluation of ChatGPT-generated medical responses: a systematic review and meta-analysis. J Biomed Inform.

[8] Fattah FH, Salih AM, Salih AM (2025). Comparative analysis of ChatGPT and Gemini (Bard) in medical inquiry: a scoping review. Front Digit Health.

[9] Dhanvijay AK, Pinjar MJ, Dhokane N, Sorte SR, Kumari A, Mondal H (2023). Performance of large language models (ChatGPT, Bing Search, and Google Bard) in solving case vignettes in physiology. Cureus.

[10] Kumari A, Kumari A, Singh A (2023). Large language models in hematology case solving: a comparative study of ChatGPT-3.5, Google Bard, and Microsoft Bing. Cureus.

[11] Koga S, Martin NB, Dickson DW (2024). Evaluating the performance of large language models: ChatGPT and Google Bard in generating differential diagnoses in clinicopathological conferences of neurodegenerative disorders. Brain Pathol.

[12] Patil NS, Huang RS, van der Pol CB, Larocque N (2024). Comparative performance of ChatGPT and Bard in a text-based radiology knowledge assessment. Can Assoc Radiol J.

[13] Wong K, Fayngersh A, Traba C, Cennimo D, Kothari N, Chen S (2024). Using ChatGPT in the development of clinical reasoning cases: a qualitative study. Cureus.

[14] Mete U (2024). Evaluating the performance of ChatGPT, Gemini, and Bing compared with resident surgeons in the otorhinolaryngology in-service training examination. Turk Arch Otorhinolaryngol.

[15] Khan MP, O'Sullivan ED (2024). A comparison of the diagnostic ability of large language models in challenging clinical cases. Front Artif Intell.

[16] Gomez-Cabello CA, Borna S, Pressman SM, Haider SA, Forte AJ (2024). Large language models for intraoperative decision support in plastic surgery: a comparison between ChatGPT-4 and Gemini. Medicina (Kaunas).

[17] Lee Y, Shin T, Tessier L (2024). Harnessing artificial intelligence in bariatric surgery: comparative analysis of ChatGPT-4, Bing, and Bard in generating clinician-level bariatric surgery recommendations. Surg Obes Relat Dis.

[18] Giannakopoulos K, Kavadella A, Aaqel Salim A, Stamatopoulos V, Kaklamanos EG (2023). Evaluation of the performance of generative AI large language models ChatGPT, Google Bard, and Microsoft Bing chat in supporting evidence-based dentistry: comparative mixed methods study. J Med Internet Res.

